# Case report and literature review: fatal cerebral fat embolism following facial autologous fat graft

**DOI:** 10.3389/fneur.2023.1180333

**Published:** 2023-08-04

**Authors:** Yawen Cheng, Gezhi Yan, Chenyang Li, Xiangning Han, Jing Shang, Suhang Shang, Jianfeng Han, Guogang Luo, Fude Liu

**Affiliations:** ^1^Department of Neurology, The First Affiliated Hospital of Xi'an Jiaotong University, Xi'an, China; ^2^Department of Neurology, HanCheng People's Hospital, Han Cheng, China; ^3^The Diagnostic Center, Shannxi People's Hospital, Xi'an, China

**Keywords:** autologous fat graft, facial injection, fat embolism, massive cerebral, infarction

## Abstract

**Background:**

Severe cerebral artery embolism is a rare complication of facial autologous fat injection. However, its incidence has markedly increased with the recent rise in facial cosmetic procedures.

**Case presentation:**

We report a 31-year-old Chinese woman who presented with unconsciousness 6 h after having undergone a facial autologous fat injection. A neurological examination revealed stupor, bilaterally diminished pupillary light reflexes, right-sided central facial palsy, and no reaction to pain stimulation of right limbs. Diffusion-weighted imaging displayed patchy hyperintense lesions in the left frontal, parietal, and temporal lobes. Magnetic resonance angiography demonstrated fat embolism in the left internal carotid artery, anterior cerebral artery, and middle cerebral artery. We immediately performed mechanical thrombectomy under sufficient preoperative preparations but failed to achieve complete recanalization. Pathological examination of the embolus confirmed the presence of adipocytes. Although we actively administered symptomatic and supportive treatments, the patient eventually died due to the progression of cerebral herniation and systemic infection.

**Conclusion:**

Due to the ineffectiveness of current treatment and the inferior prognosis, fat embolism, a severe complication of autologous fat graft, should draw the attention of both plastic surgeons and neurologists so that actions may be taken for both its prevention and treatment.

## 1. Introduction

With continuous improvements in cosmetic procedures and people's desire to pursue beauty, facial autologous fat injection has become a popular procedure due to its widespread availability, relative safety, permanent results, and low rejection rate. The procedure is widely considered to be a low-risk measure to correct soft-tissue defects with minimal discomfort to the patient (1). However, a growing number of cases of rare but severe vascular complications of facial autologous fat injection, including cerebral artery embolism, are being reported worldwide (2). As these complications have limited treatment options and carry a poor prognosis, they should draw the attention of both doctors and patients. Here, we report an uncommon case of fatal cerebral embolism after a facial autologous fat graft and put forward our recommendations to potentially prevent this devastating complication. This study was reported in agreement with the principles of the CARE guidelines (3).

## 2. Case presentation

A 31-year-old left-handed Chinese woman was admitted to our hospital due to disordered consciousness. She had undergone abdominal liposuction followed by the bilateral injection of 10.6 mL of autologous fat into the temples and tear troughs under general anesthesia 6 h ago. After the 2-h procedure, the patient was unconscious, and her right limbs were unresponsive to pain stimulation, which was attributed to a lack of recovery from general anesthesia by the cosmetic doctor. However, 1 h after the surgery, the patient had still not woken up, and her heart rate had declined to 60 bpm, which increased to 70 bpm after an intravenous bolus of 0.5 mg atropine. Before the procedure, she had been in good health and had undergone a similar procedure at the same beauty salon 5 months ago and recovered well.

**Figure 1:**
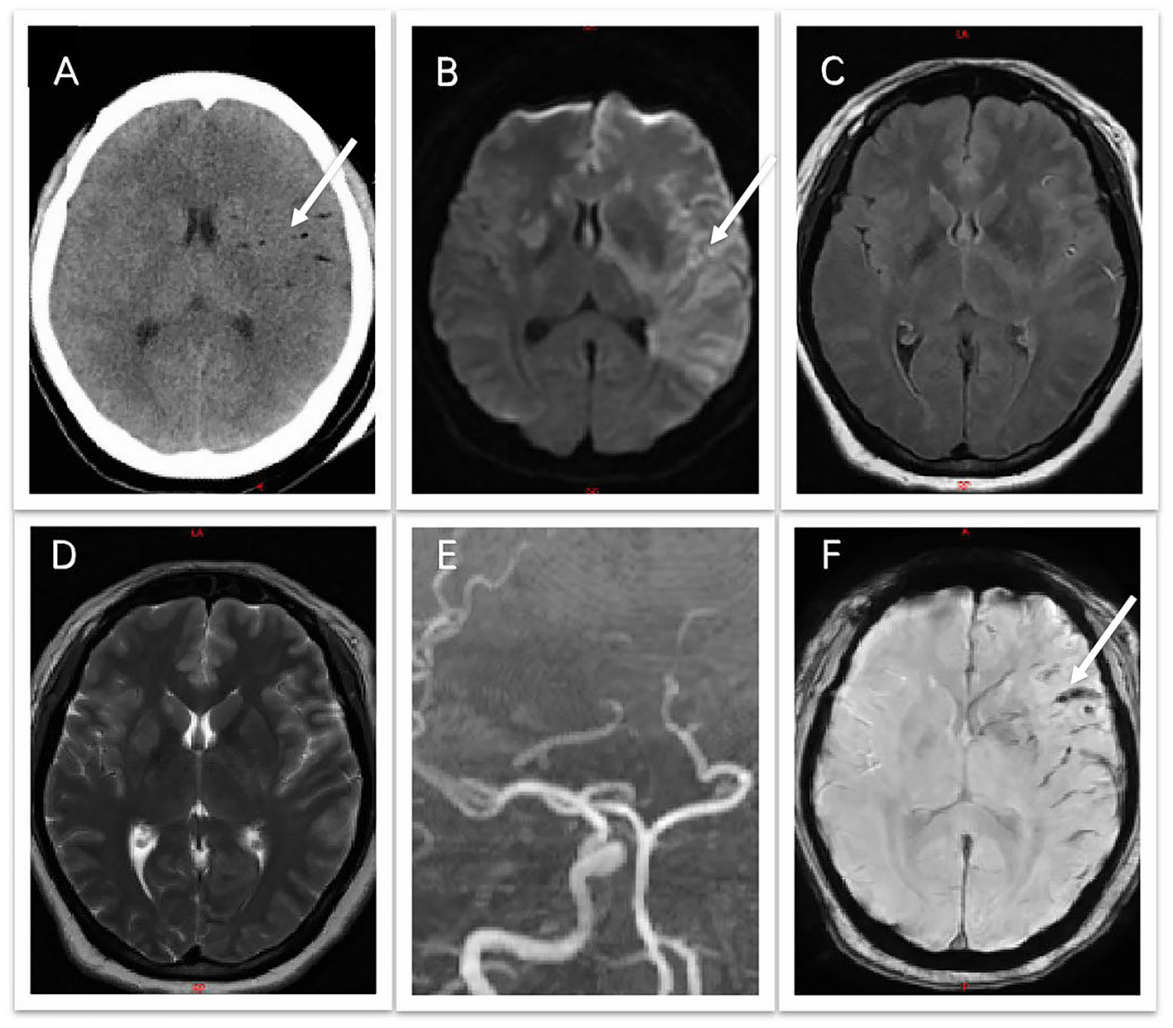
**(A)** Non-contrast computed tomography shows multiple low-density shadows along the blood vessels. **(B)** Diffusion-weighted imaging shows high-signal intensities in the left cerebral hemisphere. **(C)** Fluid attenuated inversion recovery. **(D)** T2-weighted sequences show much smaller lesions. **(E)** Magnetic resonance angiography shows occlusion of the left internal carotid artery, anterior cerebral artery, and middle cerebral artery. **(F)** Susceptibility-weighted imaging shows hypointensity along the cerebral vessels, indicating slowed blood flow caused by the fat emboli.

On admission to our hospital, the patient's vital signs were stable. A neurological examination revealed stupor, round and bilaterally equal pupils (3 mm), bilaterally diminished pupillary light reflexes, right-sided central facial palsy, no response to pain stimulation of the right limbs, and a positive Babinski sign on the right. Considering the type of operation and the patient's symptoms and signs, we suspected a cerebral fat embolism, most likely involving a vessel in the left internal carotid artery system. Although the patient had undergone abdominal liposuction, she had no rapid respiratory rate, hypoxemia, or chest CT signs such as the “blizzard sign,” ruling out pulmonary fat embolism. Cranial non-contrast computed tomography (CT) revealed multiple low-density shadows along the blood vessels (Figure 1A). Diffusion-weighted imaging (DWI) confirmed fresh infarctions in the left frontal, parietal, and temporal lobes (Figure 1B). The lesions appeared much smaller on fluid-attenuated inversion recovery (FLAIR) and T2-weighted sequences (Figures 1C, D). Magnetic resonance angiography demonstrated the occlusion of the left internal carotid artery (ICA), left anterior cerebral artery, and left middle cerebral artery (Figure 1E). No obvious hemorrhage was observed on susceptibility-weighted imaging, and the hypointense lesions along the cerebral vessels indicated slowed blood flow caused by fat emboli (Figure 1F).

**Figure 2:**
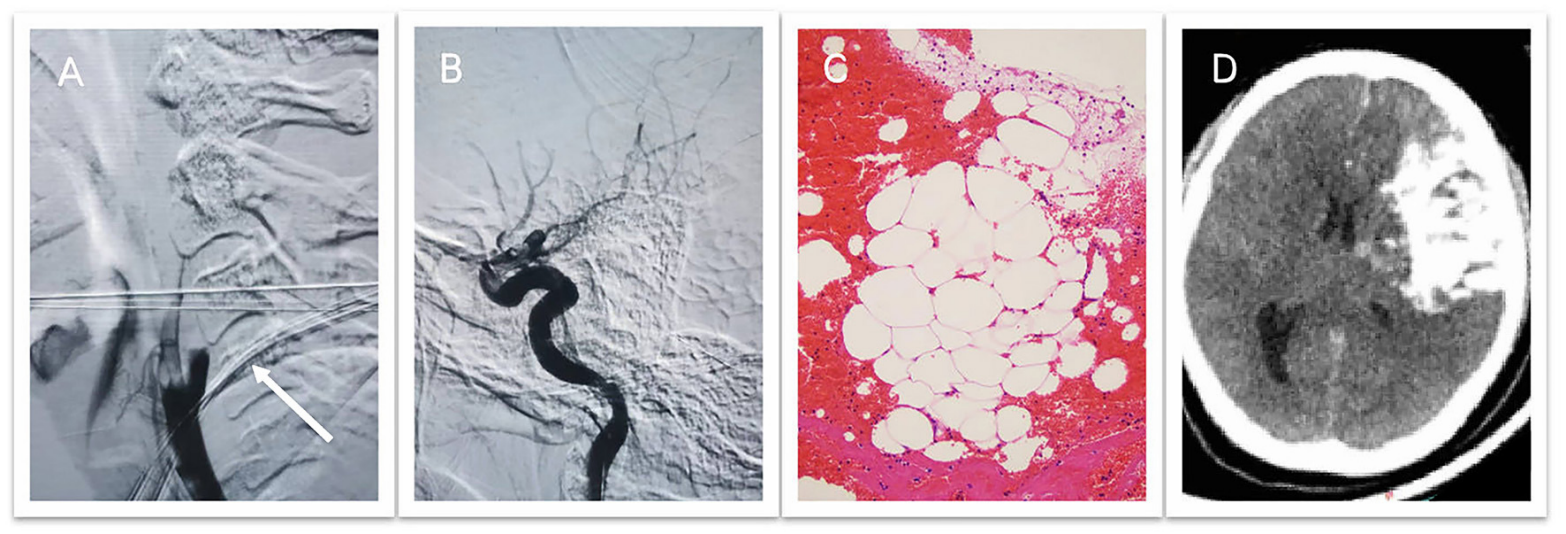
**(A)** Digital subtraction angiography confirms occlusion of the left internal and external carotid arteries. **(B)** Mechanical thrombectomy fails to achieve complete recanalization. **(C)** Pathological examination of the emboli confirms the presence of adipocytes. **(D)** Postoperative computed tomography demonstrates a large cerebral infarction with extravasation of the contrast agent in the left cerebral hemisphere and a slight rightward shift of the midline.

**Figure 3:**
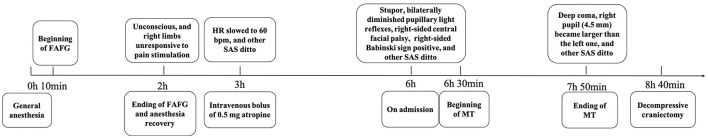
Timeline of symptoms, physical signs, and treatment. FAFG, facial autologous fat graft; HR, heart rate; SAS, symptoms and signs; MT, mechanical thrombectomy.

Owing to the mismatch between the FLAIR and DWI findings, we performed digital subtraction angiography, which confirmed the occlusion of the left ICA and external carotid artery (Figure 2A). After obtaining consent from the patient's family, we immediately performed mechanical thrombectomy (MT) using the ADAPT (a direct aspiration first pass technology) and SWIM (Solitaire FR/stent with intracranial support catheter for mechanical thrombectomy) techniques but failed to achieve complete recanalization (Figure 2B). Pathological examination of the emboli confirmed the presence of adipocytes (Figure 2C). After the operation, the patient's unconsciousness deepened, and she fell into a deep coma. Her right pupil (4.5 mm) became larger than the left one (3 mm). Immediate cranial non-contrast CT showed a large cerebral infarction in the left cerebral hemisphere and a slight rightward shift of the midline (Figure 2D). Decompressive craniectomy was promptly performed (Figure 3). Mannitol, glycerol fructose, torasemide, and albumin were administered to decrease intracranial pressure. We also actively provided symptomatic and supportive treatments, including nutritional support, antibiotic therapy, mechanical ventilation, neurotrophic factors, and cerebral protection.

Unfortunately, due to the large cerebral infarction and edema, the brain herniation progressed, compressing the hypothalamus and brainstem. The patient lost spontaneous respiration and was in a state of metabolic disorder. She also developed a severe infection of multiple systems. Eventually, on the 16th day of hospitalization, she was diagnosed with brain death.

## 3. Discussion

### 3.1. Epidemiology

Cerebral embolism as a complication of facial autologous fat graft has been increasingly reported in recent years. We performed a literature review by searching the PubMed, CNKI, and SinoMed databases for relevant articles describing any case of cerebral fat embolism following facial autologous fat graft. For PubMed, we used the following search strategy: “((fat graft^*^[Title/Abstract]) OR (fat injection[Title/Abstract]) AND (English[Filter])) AND ((stroke[Title/Abstract]) OR (cerebral fat embolism[Title/Abstract]) OR (infarction[Title/Abstract]) OR (cerebral embolism[Title/Abstract]) AND (English[Filter])).” No time limit was applied during the search. We found a total of 62 articles using the above search strategy (32 articles from PubMed, 13 from CNKI, and 17 from SinoMed). After excluding 9 reviews, 11 case reports were unrelated to cerebral embolism following facial autologous fat graft and 12 were repeated articles, and a total of 39 cases from 30 articles were included in the review (Table 1). All retrieved articles were independently reviewed by two authors (GY and YC), and eligible articles were selected by mutual agreement. According to Wang et al., the incidence of this complication has markedly increased since 2010, and most of the cases have occurred in young and middle-aged women from East Asia (2), which may be due to the aesthetic preference for facial three-dimensional contouring in this population.

**Table 1:** Cases of cerebral fat embolism following facial fat injection retrieved from three databases.

**No**.	**Year**	**Author**	**Age/ Sex**	**Country**	**Anesthesia**	**Dose**	**Injection site**	**Infarction artery/site**	**Treatment**	**Outcomes**
1.	1996	Lee et al. (5)	42/F	Korea	Local	0.5 mL	Nasolabial groove	NR	NR	NR
2	1998	Feinendegen et al. (16)	45/M	Switzerland	General	NR	Nasolabial folds, lip, and chin	MCA	NR	Aphasia
			47/F	Switzerland	Local	NR	Periorbital areas	Border zones between ACA-MCA and MCA-PCA	NR	Left-eye blindness
3	2003	Yoon et al. (17)	39/F	Korea	Local	5 mL	Glabella	ICA	Mechanical ventilation, steroids	Death
4	2004	Thaunat et al. (18)	39/M	France	Local	17 mL	Temporal, eyelids, glabella	ACA	NR	Mutism and paraplegia
5	2011	Lee et al. (19)	44/F	Korea	Intravenous	NR	Periocular	MCA	Ocular massage, carbon dioxide, oxygen therapy	Vision loss
6	2012	Lee et al. (11)	26/F	Korea	Local	NR	Face	Right parietal lobe	Steroids	NR
7	2014	Hong et al. (20)	31/F	Korea	General	NR	Glabella	MCA	Ocular massage, anterior chamber paracentesis, volume expansion	Vision loss
8	2014	Wang et al. (21)	22/F	China	General	49 mL	Temporal, frontal	ECA and ICA	Mannitol, decompressive craniectomy	Vision loss and neurological deficits mildly improved
9	2015	Chen et al. (22)	31/F	China	General	NR	Glabella	MCA	Ocular massage, anterior chamber paracentesis, volume expansion	Vision loss
10	2016	Shen et al. (23)	30/F	China	Local	44 mL	Bilateral temporal regions and the chin	ECA and ICA	Decompressive craniectomy, oxygen-free radical purging, antiplatelet aggregation	Improved mildly
11	2016	Kang et al. (24)	32/F	Korea	Local	NR	Glabella	ACA, MCA	Thrombolytic agents	Vision loss and neurological deficits mildly improved
12	2017	Lu et al. (25)	27/F	China	Local	NR	Bilateral temporal regions	Bilateral cerebral and cerebellar hemispheres	Reduce intracranial pressure, control blood pressure	Death
13	2017	Wang et al. (26)	25/F	China	NR	NR	Forehead	MCA	Ocular massage, volume expansion, antiplatelet drugs, steroids	Vision loss
			30/F	China	NR	NR	Forehead	MCA, ACA	Ocular massage, volume expansion, antiplatelet drugs, steroids	Vision loss
14	2018	Ji et al. (27)	30/F	China	NR	NR	Bilateral temporal regions	ICA, ECA	Embolectomy	Death
			23/F	China	NR	NR	Bilateral temporal regions	Left frontal and parietal lobes	Antiplatelet drugs, oxygen therapy	Vision loss
15	2018	Huo et al. (28)	33/F	China	Local	NR	Glabella	ICA, MCA	Embolectomy, decompressive craniectomy	Mild improvement
			25/F	China	Local	NR	Glabella	MCA, ACA	Embolectomy, decompressive craniectomy	Mild improvement
			24/F	China	NR	NR	Periocular region	ACA, CCA, ICA, MCA, PCA	Embolectomy	Death
			19/M	China	NR	NR	Glabella	MCA	Embolectomy, decompressive craniectomy	Mild improvement
			28/M	China	NR	NR	Glabella	NR	No	Death
16	2018	Wang et al. (29)	22/F	China	Local	NR	Temporal region	MCA, superficial temporal artery	Decompressive craniectomy	Incomplete cure
			30/F	China	Local	NR	Temporal region	Right hemisphere, superficial temporal artery	Decompressive craniectomy	Left limbs paralysis
17	2018	Liu et al. (30)	47/F	China	NR	NR	Jaw	ECA and MCA	Thrombolytic agents, antiplatelet aggregation, reduce intracranial pressure	Incomplete cure
18	2019	Wu et al. (31)	42/F	China	Local	NR	Temporal region	ICA, MCA, and ACA	Decompressive craniectomy with necrotic brain tissue resection	Mild improvement
19	2019	Renard et al. (12)	50/F	France	Local	NR	Right eye	MCA and ACA	NR	NR
20	2019	Zhou et al. (32)	22/F	China	Local	50 mL	Temporal region	ICA and MCA	Embolectomy	Incomplete cure
21	2019	Li et al. (33)	18/F	China	Local	NR	Forehead	ECA, ICA, ACA, MCA, and PCA	Mechanical ventilation, reduce intracranial pressure, control blood pressure	Death
22	2020	Huo et al. (34)	19/M	China	NR	NR	Face	ICA	Embolectomy, decompressive craniectomy, antiplatelet aggregation	Mild improvement
23	2020	Lee et al. (35)	33/F	Korea	NR	NR	Buttock	MCA	Embolectomy, antiplatelet aggregation	Cure
24	2021	Xu et al. (36)	28/F	China	NR	NR	Face	ECA, ACA, and MCA	Antiplatelet aggregation, reduce intracranial pressure, neurotrophic factor therapy	Incomplete cure
25	2021	Lu et al. (37)	35/M	China	NR	NR	Temporal region	Left frontal and parietal cortices	Intravenous thrombolysis	Mild improvement
26	2021	Liu et al. (38)	18/F	China	Local	NR	Temporal region	ECA and ICA	Decompressive craniectomy	Death
			19/F	China	Local	NR	Forehead	Parietal lobe	NR	Not improved
27	2021	Miao et al. (39)	22/F	China	General	10 mL	Temporal region	MCA, PCA	Decompressive craniectomy, mannitol, vasoactive drugs, ceftriaxone, methylprednisone	Incomplete cure
28	2021	Qian et al. (40)	28/F	China	General	77 mL	Temple, forehead, and cheeks	Frontal, temporal, parietal lobes, and the basal ganglia	Dehydration, infection prophylaxis, gastric protection, seizure prophylaxis	Mild improvement
29	2021	Liang et al. (41)	27/F	China	NR	NR	Face	Fronto-parietal junction	Hyperbaric oxygen therapy, antiplatelet aggregation	Vision loss
30	2022	Cui et al. (42)	21/F	China	NR	NR	Face	MCA	Thrombolytic agents, antiplatelet aggregation	Incomplete cure
Present case			31/F	China	General	10.6 mL	Temporal region and tear trough	ICA, MCA, and ACA	Thrombectomy, decompressive craniectomy, reduce intracranial pressure, anti-infection	Death

NR, not reported; CCA, common carotid artery; ECA, external carotid artery; ICA, internal carotid artery; ACA, anterior cerebral artery; MCA, middle cerebral artery; PCA, posterior cerebral artery.

### 3.2. Pathogenesis

Facial autologous fat graft is most commonly performed using multi-tunnel, multi-layered, and multi-point dispersed injections to uniformly fill the space between the periosteum and the muscular layer. However, this surgical approach increases the risk of puncturing blood vessels. One possible mechanism for cerebral fat embolism is that the injection device accidentally punctures the small facial veins or arteries, inducing a reflux of fat particles into the proximal large vessels under excessive pressure, which then flow down into the distal vessels (4). A small amount of fat is sufficient to cause reflux. The smallest fat volume that has been reported to cause a cerebral embolism is 0.5 mL (5).

When fat emboli enter the cerebral circulation through the venous system, the symptoms of neurological deficits may appear late and may be accompanied by some signs of pulmonary embolism such as dyspnea and tachycardia.

When fat embolism occurs via the arterial route, the fat particles inadvertently enter the superficial arteries, such as the extracranial branches of the ophthalmic artery, the facial artery, or the superficial temporal artery, and eventually reach the cerebral circulation due to hemodynamics.

Considering the graft site in our patient, we speculate that the emboli flowed back into the external carotid artery through the superficial temporal artery and then entered the ICA. It is also possible that the tear-trough modification damaged the infraorbital artery, through which the emboli could directly flow into the ophthalmic artery and then into the ICA in a retrograde fashion (6).

Some studies (7, 8) have suggested that the macroscopic pathological changes caused by fat embolism consist of occlusion and immediate ischemia in the case of arterial emboli. The microscopic pathological changes are attributable to the inflammatory cascade induced by the fat particles, which causes damage to the perivascular tissue and delayed symptoms.

### 3.3. Symptoms and diagnosis

There is no clear time boundary between early and delayed symptoms (9, 10). The severity of symptoms depends on the location and extent of the embolism. Wang et al. concluded that the infarction mainly occurred in the middle cerebral artery territory, and the most common symptoms were consciousness disorder, hemiplegia, aphasia, and vision loss (4). It is difficult to distinguish fat embolism from other types of cerebral infarction on non-contrast CT. DWI often displays sporadic hyperintense lesions resembling “a starry sky.” Lee et al. regarded magnetic resonance spectroscopy to be more helpful than DWI for identifying fat emboli (11). In our patient, the CT value of the shadows ranged from −33 HU to −120 HU, suggesting fat embolism. The diagnosis was finally confirmed by pathology.

### 3.4. Treatment and prognosis

Treatment options for cerebral fat embolism with large-vessel occlusion are limited and ineffective. Early reperfusion is crucial. Intra-arterial MT or thrombolysis is usually selected; the effect of intravenous thrombolysis is poor. Among the previously reported cases, only one patient experienced a significant improvement in neurological function after undergoing MT at 6 h and 45 min after the onset of symptoms (12). The key to a successful outcome is timely recognition and treatment. Steroids were used as well, probably to inhibit the inflammatory cascade, with unsatisfactory effects. Symptomatic treatments, including intracranial pressure reduction, anti-infection therapy, nutritional support, and brain protection, are indispensable. Even with these treatments, the prognosis is extremely poor. Among the 30 patients reported by Wang et al., four patients died, and those who survived had a poor quality of life due to irreversible hemiplegia and vision loss (2).

In our patient, the failure of MT may be attributed to the following three reasons. First, the symptoms were not recognized in time by the cosmetic doctor. The second reason is the inherent limitation of thrombectomy materials. Fat particles are liquid emboli, and currently, available stents are all open or coiled closed-loop structures, which can effectively embed a solid thrombus in their multiple layers but cannot efficiently contain liquid emboli. The third reason is that the multiple fat emboli in the distal intracranial vessels may have caused microcirculation disturbances, which may benefit little from thrombectomy. Although we performed decompressive craniectomy and administered dehydration treatment immediately after the signs of brain herniation were found and actively applied symptomatic treatments, the patient eventually died due to the progression of brain herniation and systemic infection.

### 3.5. Prevention and advice

The high fatality rate and lack of effective treatments for cerebral fat embolism highlight the need for preventive measures. We recommend the following measures to minimize the incidence of this life-threatening complication of autologous fat grafting:

Cosmetic doctors should have an in-depth knowledge of the vascular anatomy of the face to avoid inadvertently injecting fat particles into the facial blood vessels.

The fat injection site should be selected very carefully. It is generally believed that transplanted adipose tissue is more likely to survive in highly vascularized areas such as muscle though this carries a higher risk of vascular damage. Hufschmidt et al. recommend separating the cheek into two specific areas according to the depth of injection (6). Ultrasound-guided injection or preoperative epinephrine can reduce vascular damage (9).

The operation should be strictly standardized, and the vital and neurological signs of the patient should be closely monitored. The use of local anesthesia, if possible, rather than general or intravenous anesthesia is preferred. Several authors have proposed that using blunt large-caliber cannulas rather than needles may prevent the puncture of small branch vessels, which is contrary to the findings of the review by Wang et al. (4, 6, 13). Vascular complications may be reduced by injecting less than the volume required to reach the ophthalmic artery or ICA (14). Thus, multi-channel syringes with a capacity of 1 mL may be safely applied. The injection should be very slow and should be followed by pulling back the syringe under low pressure, especially in high-risk areas. Wang et al. reported that minced fat is more likely to cause embolism, and a single injection of 0.05 mL is probably safe (2).

Potent treatments need to be found for patients who do develop cerebral fat embolism. The extremely low rate of recanalization and the high risk of distal vessel embolism lead to a low benefit of MT. Some authors have treated vascular embolism after hyaluronic acid injection by intra-arterial thrombolysis using hyaluronidase combined with urokinase, which resulted in a significant improvement (15). Specific drugs for dissolving fat emboli may be a new therapeutic target for future research.

## Data availability statement

The raw data supporting the conclusions of this article will be made available by the authors, without undue reservation.

## Ethics statement

Ethical review and approval was not required for the study on human participants in accordance with the local legislation and institutional requirements. Written informed consent from the patients/participants or patients/participants' legal guardian/next of kin was not required to participate in this study in accordance with the national legislation and the institutional requirements. Written informed consent was obtained from the individual(s) and/or minor(s)' legal guardian/next of kin for the publication of any potentially identifiable images or data included in this article.

## Author contributions

The clinical data and figures were collected by CL, JS, and SS. The original manuscript was written by YC, GY, and XH and then reviewed and edited by FL, JH, and GL. All authors read and approved the final manuscript.
